# Detecting diabetic retinopathy through machine learning on electronic health record data from an urban, safety net healthcare system

**DOI:** 10.1093/jamiaopen/ooab066

**Published:** 2021-08-19

**Authors:** Omolola I Ogunyemi, Meghal Gandhi, Martin Lee, Senait Teklehaimanot, Lauren Patty Daskivich, David Hindman, Kevin Lopez, Ricky K Taira

**Affiliations:** Center for Biomedical Informatics, Charles R. Drew University of Medicine and Science, Los Angeles, California, USA; Department of Radiological Sciences, University of California, Los Angeles, California, USA; Center for Biomedical Informatics, Charles R. Drew University of Medicine and Science, Los Angeles, California, USA; Biostatistical Core, Charles R. Drew University of Medicine and Science, Los Angeles, California, USA; Department of Biostatistics, University of California Los Angeles Fielding School of Public Health, Los Angeles, California, USA; Biostatistical Core, Charles R. Drew University of Medicine and Science, Los Angeles, California, USA; Department of Surgery, Charles R. Drew University of Medicine and Science, Los Angeles, California, USA; Los Angeles County Department of Health Services, Los Angeles, California, USA; Department of Family Medicine, Charles R. Drew University of Medicine and Science, Los Angeles, California, USA; Los Angeles County Department of Public Health, Los Angeles, California, USA; Center for Biomedical Informatics, Charles R. Drew University of Medicine and Science, Los Angeles, California, USA; Department of Radiological Sciences, University of California, Los Angeles, California, USA

**Keywords:** diabetic retinopathy, machine learning, artificial intelligence, safety net providers, diabetic retinopathy diagnosis

## Abstract

**Objective:**

Clinical guidelines recommend annual eye examinations to detect diabetic retinopathy (DR) in patients with diabetes. However, timely DR detection remains a problem in medically underserved and under-resourced settings in the United States. Machine learning that identifies patients with latent/undiagnosed DR could help to address this problem.

**Materials and Methods:**

Using electronic health record data from 40 631 unique diabetic patients seen at Los Angeles County Department of Health Services healthcare facilities between January 1, 2015 and December 31, 2017, we compared ten machine learning environments, including five classifier models, for assessing the presence or absence of DR. We also used data from a distinct set of 9300 diabetic patients seen between January 1, 2018 and December 31, 2018 as an external validation set.

**Results:**

Following feature subset selection, the classifier with the best AUC on the external validation set was a deep neural network using majority class undersampling, with an AUC of 0.8, the sensitivity of 72.17%, and specificity of 74.2%.

**Discussion:**

A deep neural network produced the best AUCs and sensitivity results on the test set and external validation set. Models are intended to be used to screen guideline noncompliant diabetic patients in an urban safety-net setting.

**Conclusion:**

Machine learning on diabetic patients’ routinely collected clinical data could help clinicians in safety-net settings to identify and target unscreened diabetic patients who potentially have undiagnosed DR.

## INTRODUCTION

The most recent statistics published by the Centers for Disease Control and Prevention indicate that diabetes mellitus affects roughly 30.3 million individuals in the United States.^1^ Diabetic retinopathy (DR) is a sight-threatening complication of diabetes, affecting almost one-third of adults with diabetes over age 40.^2^ The global prevalence of DR in persons with diabetes is estimated to be 35.4%^3^ and is forecasted to almost triple during the next 45 years.^4^

Although DR is the leading cause of blindness in US adults between the ages of 20 and 74 years,^5^^,^^6^ it is treatable if detected early. Risk factors associated with DR include the length of time a person has had diabetes,^3^^,^^7–9^ high blood glucose/poor blood sugar control,^3^^,^^7–10^ high blood pressure,^3^^,^^7^^,^^8^^,^^10^ dyslipidemia/^7^ high cholesterol,^3^ pregnancy,^7^ nephropathy,^8^ obesity,^7^ inflammation,^7^ ethnicity,^7^ and insulin treatment for Type II diabetes.^8^

The American Diabetes Association recommends annual eye examinations for individuals with diabetes, although the screening schedule may be increased to 2 years after an individual has one or more annual exams indicating no DR.^11^ Adhering to an annual or even biennial screening schedule for patients with diabetes in urban, medically underserved and under-resourced areas, is complicated by a shortage of eye specialists. Teleretinal DR screening programs have been introduced in a variety of urban settings, including Los Angeles, CA and Houston, TX to address the shortage of ophthalmologists and to move diabetic eye screening from the specialist setting into the primary care setting.^12–14^

For this study, we partnered with the Los Angeles County Department of Health Services (LACDHS). LACDHS is part of the US medical safety net, which includes Federally Qualified Health Centers (FQHCs) as well as State and County hospitals that provide health care services to over 16 million patients nationally—and to roughly 2 million patients in the state of California—regardless of the patients’ health insurance status or ability to pay.^15^

LACDHS is the second-largest urban healthcare system in the United States, caring for approximately 750 000 unique patients each year,^16^ including more than 142 000 uninsured patients.^17^ Approximately 85 000 patients with diabetes were seen at LACDHS facilities between 2019 and 2020. LACDHS launched a Teleretinal Diabetic Retinopathy Screening Program and Reading Center in 2013 to address the need for annual eye screenings in the safety net setting.^13^ The program screens patients for DR from over 200 safety-net primary care clinics in Los Angeles via retinal photographs taken with a fundus camera by medical assistants or licensed vocational nurses in LACDHS primary care clinics. This eliminates the need for a separate visit to an eye care provider for those with normal images, allowing those with more advanced disease to be triaged directly to treatment/subspecialty clinics.^13^ To adequately manage resources in a setting with a large volume of diabetic patients and a relatively limited number of clinicians, retinal images from teleretinal screenings are assessed by LACDHS optometrists, with over-reads performed by a supervising ophthalmologist. Initial retinal examination rates of 37.7% across LACDHS facilities in 2012 improved to 64% in 2019 after the LACDHS Teleretinal DR Screening Program implementation. Even with these gains, identifying diabetic patients who are more likely to have DR but have not presented for teleretinal or other DR screening is a priority; 36% of eligible diabetic patients have not availed themselves of teleretinal DR screening. Frequently, patients with the greatest challenges to accessing diabetic care and DR screening are at the highest risk for disease.

Diabetic retinopathy progresses in stages, from mild nonproliferative DR (NPDR) to moderate NPDR, and from severe NPDR to proliferative DR (PDR). Visible developments in the retina as DR progresses include microaneurysms, intra-retinal hemorrhages, retinal ischemia (cotton-wool spots), venous beading, intra-retinal microvascular abnormalities, and finally, neovascularization, the proliferation or growth of fragile new blood vessels that can bleed on the retina’s inner surface.^9^ As these visible developments are identifiable in digital retinal images but not in structured clinical data, imaging phenotypes are needed for DR staging. The goal of the present work is thus to identify and target for teleretinal screening diabetic patients who: (1) may be at any stage of diabetic retinopathy and (2) have not had an LACDHS guideline-recommended annual teleretinal DR screening within 12 months (ie, patients who have no existing or updated digital retinal images), using only DR risk factor data from their clinical records. A related study examines deep learning for automatic staging and grading of diabetic retinopathy in guideline-compliant diabetic patients who have received teleretinal DR screening and is beyond the scope of the current paper.

To facilitate the identification of patients with latent or undiagnosed DR, we are developing machine learning (ML) models trained on clinical data routinely collected in the course of patient care. The end goal of this work is clinical decision support that enables timely, targeted screening of diabetic patients at the highest risk of retinopathy, thereby increasing the prevention of vision loss. Patients who have missed their annual eye examinations and are identified by the ML models as being more likely to have possible DR can be targeted to schedule their missed teleretinal DR screening or, in the case of patients with known complications such as cataracts that preclude teleretinal screening, to have an in-person eye examination with an eye care provider.

For the present article, we performed ML using structured clinical data from diabetic patients seen at LACDHS for either teleretinal screening or in-person eye examinations between January 1, 2015 and December 31, 2017, obtaining data on 40 631 unique patients and 30 risk factors for DR. As an external validation set for the ML models, we collected data from a set of 9300 patients who were seen at LACDHS between January 1, 2018 and December 31, 2018, and who were completely distinct from the patients whose data were used to train and initially test the ML models.

## METHODS

Institutional review board approval to use clinical data for the study was obtained from the Charles R. Drew University of Medicine and Science under IRB#: 16-10-2491-03.

### Data source and description

LACDHS uses a Cerner electronic health record (EHR) system, ORCHID/Millenium Powerchart which feeds data into data warehouses accessible to the USC and UCLA CTSIs. We utilized the services of both the UCLA and USC CTSI’s Biomedical Informatics teams to obtain data for the study. To establish counts of eligible diabetic patients for our study, we utilized the UCLA CTSI’s Los Angeles Data Resource, creating a search for Type 1 and Type II diabetic patients 18 years or older seen at LACDHS between 2015 and 2017 who received an eye examination. We then provided ICD-9 and ICD-10 codes corresponding to relevant risk factors for DR to the USC CTSI for retrieval of the relevant cases and obtained structured clinical data from them for the study. Our inclusion/search criteria were for patients 18 years or older with Type I or Type II diabetes, who received an eye examination at County facilities between January 1, 2015 and December 31, 2017. Exclusion criteria included patients under the age of 18, patients who had only gestational diabetes, and patients who did not receive an eye examination for DR. There were 40 631 patient records retrieved from ORCHID with records containing 31 variables, including DR diagnosis, as listed in Table 1. Variables retrieved for the study correspond to known DR risk factors documented in the biomedical literature (eg, duration of diabetes,^3^^,^^7–9^ high blood glucose/poor blood sugar control,^3^^,^^7–10^ high blood pressure,^3^^,^^7^^,^^8^^,^^10^ dyslipidemia/^7^ high cholesterol,^3^ pregnancy,^7^ nephropathy,^8^ obesity,^7^ inflammation,^7^ ethnicity,^7^ and insulin treatment for Type II diabetes^8^) as well as suggestions from clinician experts regarding variables routinely collected in the EHR that might address micro and macrovascular complications of diabetes.

**Table 1.** Clinical variables for patients with diabetes obtained from LACDHS EHR system

Socio-demographic variables		
Age^a^	Race	Ethnicity^a^
Sex^a^	Marital status	Insurance status
General health overview		
Diabetes diagnosis date^a^^,^^b^	Date of last eye examination^b^	Pregnancy status
Previous diabetic retinopathy treatment	Smoking status^c^	Insulin dependence^a^
Clinical measurements		
Body mass index	Diastolic blood pressure^a^	Fasting glucose level^c^
Blood urea nitrogen^a^	Systolic blood pressure^a^	HDL
Hemoglobin^a^	Hemoglobin A1C^a^	Triglycerides^a^
Comorbid conditions		
Peripheral vascular disease	Hypertension	Stroke^a^
Depression	Obesity	Nephropathy^a^
Dyslipidemia	Neuropathy^a^	Erectile dysfunction
Condition of interest		
Diabetic retinopathy diagnosis		

Included (or derived variable included) in 14-variable feature subset used for ML.

Converted to “duration of diabetes in years” + and “time since last eye exam in months”.

Dropped due to >35% missing data.

Of the 40 631 patient records obtained, 12 633 records (31.1%) represented diabetic patients with a diagnosis of DR and 27 998 (68.9%) represented diabetic patients with no DR. This represents a *class imbalance*: the majority of available data involves cases in which patients did not have DR.

Variables with more than 35% missing data were dropped from use for ML. Date variables such as “diabetes diagnosis date” and “date of last eye examination” were transformed into the numerical variables “duration of diabetes in years” and “time since the last eye exam in months.” Although only a small proportion of patients had previous retinopathy treatment, since it is highly correlated with the outcome variable (most people who have had previous retinopathy treatment currently have DR), it was dropped from use for ML. The final set of 14 predictors utilized for ML for the study, including “duration of diabetes in years,” are listed in Table 1.

For an external validation set, through the USC CTSI, we acquired data from ORCHID for 9300 Type I and Type-II diabetes patients who were seen at LACDHS between January 1, 2018 and December 31, 2018, and whose data were not used to train or test the ML models.

### Feature subset selection

We performed feature subset selection on the variables from Table 1: (1) to eliminate variables that were not highly predictive of DR in our data set and (2) to make the final models more clinician-friendly by determining a smaller subset of predictors for DR to be input into a DR identification tool by a clinician user. Feature subset selection was performed by first establishing a lower and upper bound on the number of variables that meaningfully predict DR and then by identifying which variables should be in the feature subset.

### Classification methods

We handled missing data using k-nearest neighbor imputation techniques with a k of 9. Numeric variables were normalized prior to use in ML. Five supervised learning models were applied: (1) a random forest (RF) model; (2) a support vector machine (SVM) model; (3) extreme gradient boosting (XGBOOST); (4) an ensemble of four stacked classifiers with random forest, gradient boosting machines, and artificial neural networks in the top layer and gradient boosting machines in the bottom layer; and (5) deep learning with a deep neural network (DNN).

To address the class imbalance, two case sampling methods were applied: (1) majority class undersampling; and (2) the synthetic minority over-sampling technique (SMOTE).^18^ In total, ten modeling-sampling combinations were developed: (1) RF with undersampling; (2) RF with SMOTE; (3) SVM with undersampling; (4) SVM with SMOTE; (5) XGBOOST with undersampling; (6) XGBOOST with SMOTE; (7) ensemble model with undersampling; (8) ensemble model with SMOTE; (9) DNN with undersampling; and (10) DNN with SMOTE. We assessed classifiers on all 14 predictor variables identified through feature subset selection.

We reserved a random selection of 33% of the 2015-2017 data set, a total of 13 408 cases, as a test set. Using the caret^19^ package in R,^20^ we performed 10-fold cross-validation with parameter tuning on the remaining 67% of the data set (27 223 cases), using the first four classifiers described above. For deep learning with DNNs, we used the sklearn^21^ package in Python^22^ and developed multilayer deep neural networks using the hyperbolic tangent activation (tanh) function and a stochastic gradient descent (SGD) optimizer. For each of the ten modeling-sampling combinations specified above, the best model from 10-fold cross-validation was selected and saved, with the metric for judging the best model being the area under the ROC curve (caret also allows accuracy or Kappa to be used in place of AUC to judge the best model). Data preprocessing methods such as majority class undersampling and SMOTE were applied to the training sets derived from the 27 223 cases. The ten best models saved from the cross-validation process were assessed on the test set and then on the external validation set. Analyses were performed in R, using the caret and the VIM^23^ packages as well as in Python.

### Comparisons with similar studies

Most of the related work on ML to detect DR from clinical or public health data has focused on smaller study populations involving fewer than 1300 diabetic patients, and in some cases, more homogenous study populations, with the resulting AUCs ranging from 0.71 to 0.839.^24–29^ We compare our results with some of these studies.

## RESULTS

### Demographics

Table 2 gives an overview of the socio-demographic characteristics collected for the 40 631 diabetic patients in the training and test set population. Missing data are included for each category.

**Table 2.** Study population characteristics (*N* = 40 631)

Participant characteristics	*N* (%)
Age in years	
Mean (SD)	57.5 (10.5)
Missing values	0 (0.0)
Gender	
Male	23 242 (57.20)
Female	17 389 (42.80)
Missing values	0 (0.0)
Ethnicity	
Hispanic or Latino	28 040 (69.01)
Not Hispanic or Latino	9264 (22.80)
Missing values	3327 (8.19)
Race	
American Indian or Alaska Native	135 (0.33)
Asian or Asian American	2626 (6.46)
Black or African American	3324 (8.18)
More than one race	17 612 (43.35)
Native Hawaiian or other Pacific Islander	81 (0.20)
White	4269 (10.51)
Missing values	12 584 (30.97)
Marital status	
Single	13 940 (34.31)
Married	13 807 (33.98)
Divorced	1397 (3.44)
Widowed	1407 (3.46)
Separated	861 (2.12)
Missing values	9219 (22.69)
Insurance provider	
Medicaid	24 117 (59.36)
Medicare	4274 10.52)
Private	5569 (13.71)
Self-pay	4887 (12.03)
CHAMPUS	1 (0.00)
Other	98 (0.24)
Missing values	1685 (4.15)
Diagnosis of retinopathy	
Yes	12 633 (31.09)
No	27 998 (68.91)
Missing values	0 (0.0)

### Univariate analyses

Table 3 lists the results from using individual logistic regression models for each variable to determine its relationship to the presence or absence of DR. Many of the variables are very significant because of the large sample size (*n* = 40 631). Odds ratios and 95% confidence intervals were not calculated for nonsignificant predictors.

**Table 3.** Univariate analyses of potential predictor variables

Variable	P-value for testing significanceas predictor	Odds ratio	95% confidenceinterval	Interpretation
Age	.0003	1.00	1.00–1.01	Older, higher risk
Marital status (single vs. married)	.08			
Sex	<.00001	1.54	1.48–1.61	Males are higher risk
Ethnicity	<.00001	0.63	0.60–0.67	Not Hispanic or Latino is lower risk
Duration of diabetes (years)	<.00001	1.08	1.07–1.08	Longer duration, higher risk
Pregnancy status	.20			
Insulin dependence	<.00001	3.42	3.28–3.58	Insulin dependence, higher risk
Time since last eye exam (months)	<.00001	0.98	0.98–0.98	Longer time since last eye exam, lower risk
Peripheral vascular disease	<.00001	2.85	2.42–3.34	Peripheral vascular disease, higher risk
Hypertension	.024			
Systolic blood pressure	<.00001	1.03	1.02–1.03	Higher systolic blood pressure, higher risk
Diastolic blood pressure	.00022	1.01	1.00–1.01	Higher diastolic blood pressure, higher risk
Depression	.002	0.90	0.83–0.96	Depression, lower risk
Obesity	<.00001	0.63	0.60–0.66	Obesity, lower risk
BMI	<.00001	0.97	0.96–0.97	Higher BMI, lower risk
Stroke	<.00001	1.82	1.64–2.01	Stroke, higher risk
Nephropathy	<.00001	3.73	3.53–3.94	Nephropathy, higher risk
Erectile dysfunction	<.00001	1.34	1.19–1.51	Erectile dysfunction, higher risk
Neuropathy	<.00001	2.27	2.14–2.40	Neuropathy, higher risk
Dyslipidemia	<.00001	0.65	0.62–0.68	Dyslipidemia, lower risk
Insurance	.05			
BUN	<.00001	1.09	1.09–1.09	Higher blood urea nitrogen, higher risk
HDL	.16			
Hemoglobin	<.00001	0.73	0.72–0.74	Higher hemoglobin, lower risk
Hemoglobin A1c	<.00001	1.24	1.22–1.25	Higher hemoglobin A1C, higher risk
Triglycerides	.00002	1.00	1.00–1.00	Higher triglycerides, lower risk

After completing the univariate analysis summarized in Table 3, we dropped three potential variables from use in our modeling efforts: obesity, BMI, and time since last eye exam in months. Time since last eye exam in months was dropped because the univariate analysis shows that the longer an LACDHS patient waits to be examined for DR, the less likely the patient is to have DR. While this may seem counterintuitive, this is because LACDHS patients with moderate or worse DR are seen several times a year for eye examinations, in accordance with local clinical guidelines, when compared to patients who have mild DR or no DR, who are seen just once a year. For the present study, we are trying to determine the DR risk of patients who have *self-excluded* from DR screening; we do not want a ML model to automatically assume that these patients are at lower risk for DR because they have not been examined recently, so that variable was dropped. Obesity and BMI were dropped from our current modeling effort because the univariate analysis showed that LACDHS patients who are obese or have higher BMI are less likely to develop DR. As these findings run counter to published knowledge on the relationship between obesity or high BMI and DR, we have excluded these two variables from modeling. Our study population includes patients with both Type 1 and Type 2 diabetes. While the vast majority of patients in our study have Type 2 diabetes and are overweight or obese, patients with Type 1 diabetes tend to be leaner and to progress to diabetic retinopathy more quickly. This likely influenced the analysis showing a higher BMI or a diagnosis of obesity as indicating a lower likelihood of having diabetic retinopathy, even though the literature points to high BMI as a risk factor for DR. We thus removed these two variables until such a time as we have an opportunity to study the impact of BMI and obesity further in different diabetic patient populations.

### Feature subset selection

**Figure 1.**
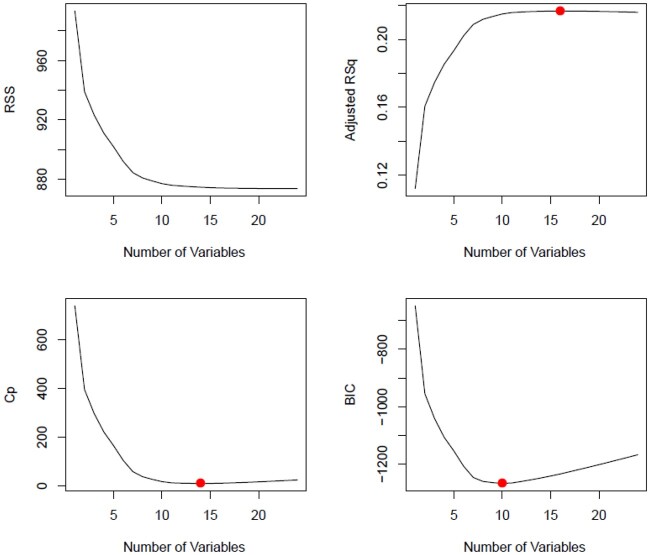
Optimal number of variables/features using various metrics.

To find the optimum number of features for modeling for our data set, we applied best subset selection regression along with *R*^2^, adjusted *R*^2^, Mallow’s C_P_ (equivalent to the Akaike information criterion), and Bayesian information criterion assessments. We found that the optimum number of features required for our model has a range from 10 to 15, as shown in Figure 1. We then performed forward stepwise regression and backward stepwise regression in order to find those features. Beginning with 10 features and adding features incrementally until we got to 15 features, we ran models and compared their results using sensitivity, specificity, and the area under the ROC curve as assessment metrics. We observed that there was minimal difference between the 15-feature model and the 14-feature model and therefore selected the 14-feature model for our data set.

**Figure 2.**
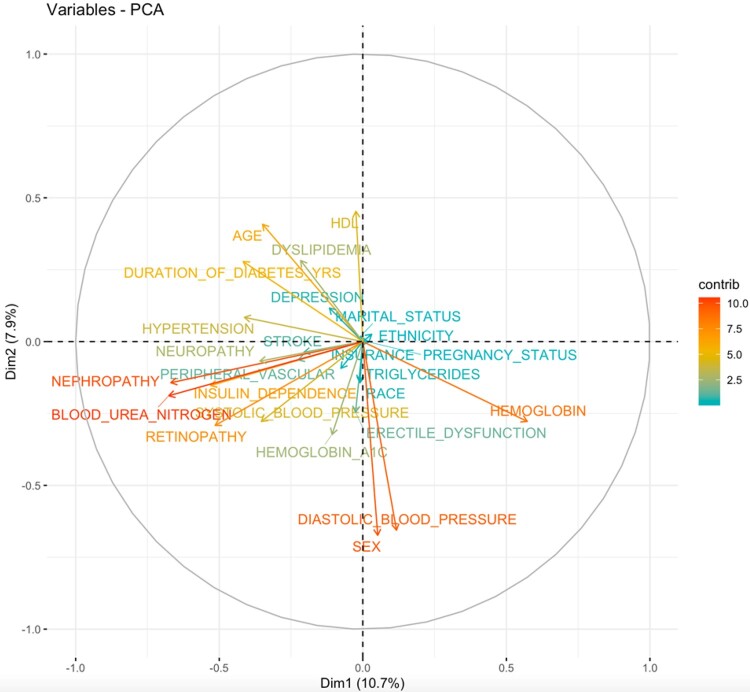
Variable correlations—principal components analysis.

In order to determine the most significant predictors of DR, potential predictors from Table 3 that exhibited a *P*-value of <.01 from the univariate analyses (excluding the three dropped variables: obesity, BMI, and time since last eye exam in months) were included in a hierarchical stepwise switching algorithm to find the best set of fourteen variables in a multivariate logistic regression. The fourteen most significant predictors of DR from our data set are:

1. Insulin dependence = “Y” (odds ratio (OR) for DR: 2.6, 95% confidence interval for OR: 2.4–2.8);
2. BUN (OR for DR: 1.04, 95% confidence interval for OR: 1.04–1.05);
3. Systolic blood pressure (OR for DR: 1.03, 95% confidence interval for OR: 1.02–1.03);
4. Neuropathy = “Y” (OR for DR: 1.63, 95% confidence interval for OR: 1.48–1.79);
5. Hemoglobin A1c (OR for DR: 1.20, 95% confidence interval for OR: 1.17–1.22);
6. Hemoglobin (OR for DR: 0.82, 95% confidence interval for OR: 0.8–0.84);
7. Sex = “M” (OR for DR: 1.95, 95% confidence interval for OR: 1.79–2.13);
8. Ethnicity=“Not Hispanic or Latino” (OR for DR: 0.66, 95% confidence interval for OR: 0.6–0.72);
9. Nephropathy = “Y” (OR for DR: 1.29, 95% confidence interval for OR: 1.16–1.45);
10. Duration of diabetes (OR for DR: 1.05, 95% confidence interval for OR: 1.03–1.06);
11. Triglycerides (OR for DR: 1.0, 95% confidence interval for OR: 1.0–1.0);
12. Stroke = “Y” (OR for DR: 1.4, 95% confidence interval for OR: 1.18–1.65);
13. Diastolic blood pressure (OR for DR: 0.99, 95% confidence interval for OR: 0.98–0.99); and
14. Age (OR for DR: 0.99, 95% confidence interval for OR: 0.99–1.0)

### Classification results

Using the 14-variable feature subset found to be most predictive, we performed 10-fold cross-validation with the ten classifier-sampling strategy combinations previously described in the Methods section on the data set. Next, we applied the best models for each classification method to the test set and then to the external validation set from 2018. For the application of the best models to the external validation set, we assessed sensitivity, specificity, positive predictive value (PPV), negative predictive value (NPV), accuracy, the Kappa statistic, and AUC.

Table 4 lists the classification results, using majority class undersampling and SMOTE respectively, on the 14 predictor variables to the test set and then to the validation set. The highest AUC and sensitivity values are bolded. We also created models on all 24 predictors; full results for those are included in a Supplementary material and show that an xgboost model with majority class undersampling produced the highest AUC on the test set (AUC = 0.813, sensitivity = 72%, specificity = 75.43%), while a deep neural network with majority class undersampling produced the highest AUC on the external validation set (AUC = 0.805, sensitivity = 71.1%, specificity = 73.9%).

**Table 4.** 14 variable model performance on test and validation sets

	RF under	XGBOOST under	SVM under	Ensemble model under	DNN under
Model performance on 14 Variables with majority class undersampling on test set					
Sensitivity (%)	71.52	70.85	72.81	70.68	**73.55**
Specificity (%)	73.51	74.61	72.58	74.96	72.77
PPV (%)	54.97	55.79	54.56	56.07	54.98
NPV (%)	85.09	84.99	85.52	84.97	85.88
Accuracy (%)	72.89	73.44	72.65	73.63	73.01
Kappa statistic	0.416	0.423	0.4158	0.426	0.4240
AUC	0.799	0.800	0.798	0.803	**0.806**
Model performance on 14 variables with majority class undersampling on external validation set					
Sensitivity (%)	69.06	66.78	70.00	67.38	**72.17**
Specificity (%)	76.01	77.35	75.24	77.09	74.20
PPV (%)	44.45	45.05	44	44.98	43.75
NPV (%)	89.83	89.34	90.02	89.47	90.55
Accuracy (%)	74.49	75.05	74.1	74.98	73.76
Kappa statistic	0.3756	0.3759	0.3728	0.3769	0.3756
AUC	0.791	0.792	0.794	0.794	**0.8**

	RF SMOTE	Xgboost SMOTE	SVM SMOTE	Ensemble model SMOTE	DNN SMOTE
Model performance on 14 variables with SMOTE on test set					
Sensitivity (%)	62.23	49.89	61.51	63.93	**72.91**
Specificity (%)	80.93	86.55	82.39	79.84	72.78
PPV (%)	59.60	62.65	61.23	58.92	54.77
NPV (%)	82.57	79.25	82.56	83.04	85.59
Accuracy (%)	75.1	75.13	75.89	74.89	72.82
Kappa statistic	0.4264	0.3859	0.4384	0.4278	0.4189
AUC	0.8	0.781	0.797	**0.803**	**0.8**
Model performance on 14 variables with SMOTE on external validation set					
Sensitivity (%)	57.24	46.02	58.13	60.21	**70.63**
Specificity (%)	83.43	87.66	84.36	82.36	74.54
PPV (%)	48.98	50.90	50.82	48.68	43.55
NPV (%)	87.53	85.38	87.88	88.16	90.13
Accuracy (%)	77.73	78.6	78.66	77.54	73.69
Kappa statistic	0.3834	0.3489	0.404	0.3921	0.3690
AUC	0.79	0.777	0.790	**0.795**	0.794

## DISCUSSION

### Overview

Overall, our results show that the classifiers developed were able to predict DR relatively well; the data preprocessing methods used were able to overcome standard classifiers’ tendency to simply predict the majority class (ie, no DR), although models that utilized majority class undersampling produced better sensitivities on the test and external validation sets. Models that utilized SMOTE had better results for specificity overall on both the test and external validation sets. Multivariate analyses showed that the 14 most significant predictors of DR were insulin dependence, BUN, systolic blood pressure, neuropathy, hemoglobin A1C, hemoglobin, sex, ethnicity, nephropathy, duration of diabetes, triglycerides, stroke, diastolic blood pressure, and age. These analyses were utilized in developing parsimonious ML models that still provide good sensitivities, specificities, and AUCs without requiring all potential DR risk factors collected. Although our ML classifiers trained on 24 variables performed slightly better than ML classifiers trained on 14 variables, we focus our discussion below on the ML classifiers trained on 14 variables, since a goal of ours is to develop tools for clinicians based on our ML models that do not require an inordinate amount of time on data entry.

### Comparison of results to those from other studies

We compare our current results to those obtained by four other studies attempting to identify patients with undiagnosed DR from clinical or public health data.

1. Korea National Health and Nutrition Examination Surveys (KNHANES) study: ^26^
  - Training set (*n* = 490)
  - Test set (*n* = 163): AUC = 0.83, sensitivity=71%, specificity=75.8%, model=SVM
  - Validation set (*n* = 562): AUC=0.82, sensitivity=72.1%, specificity=76%, model=penalized logistic regressionThis study utilized public health records of 490 individuals from Korea for machine learning to assess DR, learning penalized logistic regression, SVM, ANN, and Random Forest models. The study authors utilized between 12 and 19 predictor variables for different ML models and assessments. Results for their best models on test and validation sets are shown above.
2. Taiwan study: ^27^
  - Total records (*n* = 212), train-test-validation split = 60%, 20%, 20%
  - Test set: AUC = 0.744, accuracy = 81.7%, model = SVM
  - Validation set: AUC = 0.801, accuracy = 82.2%, model = SVMThis study utilized clinical data from 212 patients at a private hospital in Taiwan to study DR diagnosis using a variety of machine learning models, including SVMs, ANNs, and Decision Trees. Results for the best models on their test and validation sets are shown above.
3. US National Health and Nutrition Examination Surveys (NHANES):^28^
  - Total records (*n* = 266)
  - AUC = 0.74, precision = 22%, NPV = 99% (results provided are assumed to be on the training set)This study included an assessment of DR in 266 individuals with previously undiagnosed diabetes sampled from the 2005 to 2008 versions of NHANES public health data. Authors did not make a distinction between training, test, and external validation sets, so the results provided above are assumed to be on the training set. Sensitivities and specificities were not reported.
4. Iran study: ^29^
  - Total records (*n* = 133)
  - AUC = 0.804, sensitivity = 76%, specificity = 80.4%, model = deep neural network (results provided were from 10-fold cross-validation on the training set)This study evaluated deep neural networks with recursive feature elimination on risk factor data from 133 Iranian diabetic patients, going from 24 to 14 risk factors. Data were originally collected for assessment of diabetes complications from hospitals in Khorramabad, Iran and reused in this study for ML on DR. No test set or external validation set details for DR were provided.

### Assessment of classification results on test and external validation sets

For the models using 14 variables, the classifier with the best AUC on the test set was a deep neural network model using majority class undersampling, with an AUC of 0.81, the sensitivity of 73.55%, and specificity of 72.77%. The model with the highest sensitivity for detecting DR on the test set was also the deep neural network classifier.

The classifier with the best AUC on the external validation set was a deep neural network model using majority class undersampling, with an AUC of 0.8, the sensitivity of 72.17%, and specificity of 74.20%. The model with the highest sensitivity for detecting DR on the external validation set was also the deep neural network classifier.

Improvements in sensitivity came with the trade-off of decreases in specificity. The models did well on the external validation set, which was composed of a distinct set of patients seen at least one year after the patients whose data were used for training and testing, with no overlap among the three groups. This bodes well for the models’ ability to generalize to previously unseen diabetic patients within the LACDHS setting.

Our ML results compared well to those presented in similar studies, which utilized both public health (Korea, US) and hospital data (Taiwan, Iran). On external validation sets, which provide a sense of how well a model might generalize to previously unseen patients, our deep learning model on 14 variables with undersampling produced similar AUCs (0.8) and sensitivities (72.17% vs 72.1%), lower specificity (74.2% vs 76%) and lower accuracy (73.76% vs 75.2%) than the KNHANES study and a similar AUC (0.8 vs 0.801) but lower accuracy (73.01% vs 82.2%) (sensitivity and specificity were not reported) than the study using data from a private hospital in Taiwan. Our training, test and external validation data sets were more than sixty times the sizes of the comparable data sets utilized for the KNHANES and Taiwan studies. We did not compare our results to those from studies reporting only 10-fold cross-validation results on the training set, as that does not give a good indication of model generalizability; we were able to produce ML models with AUCs of 1, sensitivities of 1, and specificities of 1, using 10-fold cross-validation on our training set, indicating model overfit.

Since the clinical goal of DR detection from EHR data using ML is to identify patients who may have developed DR but are unaware of the fact because they have not had their recommended annual eye examination, classifiers that are able to maintain good sensitivity are critical. However, we also would like to utilize methods that achieve a minimum AUC of 0.8, which means that there cannot be a precipitous drop in specificity. For our data set, majority class undersampling techniques were more successful than minority class oversampling approaches in terms of improving sensitivity while maintaining decent specificity. Classifiers coupled with minority class oversampling techniques achieved higher specificities but poorer sensitivities in detecting DR.

This study marks significant progress in the search for useful diagnostic models for detecting probable DR solely from clinical, nonimage data. Earlier, we conducted a pilot ML study that used 8 variables documented in EyePACS (EyePACS, LLC) teleretinal screening records for 27 116 diabetic patients, for which the highest classifier AUC achieved was 0.745.^30^ That study was limited by the fact that detailed, relevant data related to clinical measurements and comorbid conditions from the EHR, such as BUN, blood pressure, nephropathy, neuropathy, and triglycerides were not documented within the EyePACS records available to us. This study of 40 631 diabetic patients seen in a safety net healthcare system incorporates a wider range of relevant risk factors available from the EHR and is the largest thus far focusing on DR prediction from nonimage data.

Duration of diabetes is a well-known risk factor for DR. A limitation of our study is that the diagnosis date for diabetes recorded in the LACDHS EHR represents when a patient was confirmed to have diabetes by physicians within the LACDHS system and not necessarily when the patient first received the diagnosis (if made outside the LACDHS system). Therefore, if the initial diagnosis occurred outside the LACDHS system it was not included in the current analysis, resulting in artificial truncation of duration of diabetes in our data set. This could possibly be addressed in the future by using natural language processing to extract information on a patient’s self-reported duration of diabetes based on tests performed at other clinical sites and relayed to their LACDHS providers. Also, our study by necessity only included diabetes patients who had received an eye examination. There may thus be an undersampling or oversampling of DR in our study sample, depending on the characteristics of the population who did not receive eye examinations.

To our knowledge, our work is the largest study of diabetic retinopathy risk assessment from EHR data in a US population, with data from over 27 000 diabetic patients utilized for training ML classifiers. The ML models presented are intended to be used in California urban safety-net settings, which cater to uninsured and underinsured patients, and in which shortages of ophthalmologists have necessitated the use of teleretinal screening as the primary means of diabetic retinopathy detection in diabetes patients. The primary intended end users of tools based on the machine learning models we have developed would be (1) primary care physicians in the safety net who are seeking to convince their diabetic patients that have not yet been assessed for the risk of diabetic retinopathy to receive teleretinal screenings and (2) clinicians associated with teleretinal screening programs that are seeking to reach out to high-risk patients who have fallen out of compliance with guidelines urging annual screening.

Overall, the results observed in our study are encouraging. As a major target for quality improvement within LACDHS is diabetic patients’ compliance with LACDHS annual screening guidelines, in the near term, we will apply the ML models to data from over 31 000 LACDHS diabetic patients who have not received a teleretinal DR screening in over a year, due to a combination of patient self-exclusion and COVID-19 pandemic-related teleretinal DR screening service pauses. The models will help guide the prioritization of outreach to diabetic patients who are not guideline-compliant and provide a real-world assessment of the ML models’ utility in a major safety net setting.

## CONCLUSION

For individuals at risk of DR, timely screening, diagnosis and treatment are keys to preventing vision loss. We have presented methods that will further our goal of creating an assessment tool that can assist clinicians in targeting diabetic patients in medically underserved and under-resourced settings who have not completed an annual eye examination but may have as yet undiagnosed DR. The ML methods developed to illustrate that it is possible to identify at-risk patients using routinely collected clinical data from the EHR.

## SUPPLEMENTARY MATERIAL

Supplementary material is available at *Journal of the American Medical Informatics Association* online.

## CONTRIBUTORS

O.O. conceived, designed, and coordinated the study as well as the manuscript writing. O.O. and M.G. performed data analyses, including developing and evaluating different machine learning approaches. M.L. performed univariate analyses and contributed to feature subset selection methods. S.T. performed demographic data analyses and missing data calculations. K.L. researched and contributed data on related studies. L.D. facilitated data acquisition for the study and provided clinical expertise in ophthalmology. D.H. provided guidance on safety net and behavioral considerations for the study. R.T. gave suggestions on machine learning analyses to be included in the study. All authors contributed to the drafting of the manuscript.

## Supplementary Material

ooab066_Supplementary_Data

## Data Availability

The data underlying this article were acquired from an urban safety net healthcare setting and will be shared on reasonable request with approval from that safety net healthcare setting. Please contact corresponding author lolaogunyemi@cdrewu.edu for details.
