# Supplementary material for: Detecting diabetic retinopathy through machine learning on electronic health record data from an urban, safety net healthcare system
Source: JAMIA Open. 2021 Aug 19;4(3):ooab066. doi: 10.1093/jamiaopen/ooab066 (PMC8374369; doi:10.1093/jamiaopen/ooab066)
Supplement: ooab066_Supplementary_Data [file ooab066_supplementary_data.docx]

**Supplemental File: Machine Learning Results on 24 Variables**

| Socio-demographic variables | | |
| --- | --- | --- |
| Age^+^ | Race | Ethnicity^+^ |
| Sex^+^ | Marital Status | Insurance Status |
| General Health Overview | | |
| Duration of Diabetes in Years | Insulin Dependence^+^ | Pregnancy Status |
| Clinical Measurements | | |
| Blood Urea Nitrogen^+^ | Diastolic Blood Pressure^+^ | HDL |
| Hemoglobin^+^ | Systolic Blood Pressure^+^ | Triglycerides^+^ |
| Hemoglobin A1C^+^ |  |  |
| Co-morbid Conditions | | |
| Peripheral Vascular Disease | Hypertension | Stroke^+^ |
| Depression | Neuropathy^+^ | Nephropathy^+^ |
| Dyslipidemia | Erectile Dysfunction |  |
| Condition of Interest | | |
| Diabetic Retinopathy Diagnosis |  |  |

The table below (Table 5) presents results of machine learning on the 24 predictor variables from the table above.

**Table 5.** 24 Variable Model Performance on Test and Validation Sets

| Model Performance on 24 Variables with Majority Class Undersampling on Test Set | | | | | |
| --- | --- | --- | --- | --- | --- |
|  | **RF Under** | **XGBOOST Under** | **SVM Under** | **Ensemble Model Under** | **DNN Under** |
| Sensitivity | 70.90% | 72.00% | 73.01% | 69.20% | 73.00% |
| Specificity | 75.74% | 75.43% | 73.37% | 76.79% | 74.46% |
| PPV | 56.92% | 56.99% | 55.35% | 57.41% | 56.38% |
| NPV | 85.20% | 85.63% | 85.74% | 84.65% | 85.91% |
| Accuracy | 74.23% | 74.36% | 73.25% | 74.43% | 74.00% |
| Kappa statistic | 0.437 | 0.4423 | 0.4265 | 0.4354 | 0.4392 |
| AUC | 0.809 | 0.813 | 0.802 | 0.812 | 0.811 |
| Model Performance on 24 Variables with Majority Class Undersampling on External Validation Set | | | | | |
|  | **RF Under** | **XGBOOST Under** | **SVM Under** | **Ensemble Model Under** | **DNN Under** |
| Sensitivity | 69.65% | 68.56% | 70.59% | 63.47% | 71.08% |
| Specificity | 76.39% | 76.79% | 74.99% | 81.09% | 74.93% |
| PPV | 45.06% | 45.09% | 43.97% | 48.27% | 44.08% |
| NPV | 90.05% | 89.78% | 90.17% | 88.87% | 90.31% |
| Accuracy | 74.92% | 75% | 74.03% | 77.26% | 74.09% |
| Kappa statistic | 0.3846 | 0.3818 | 0.374 | 0.4001 | 0.3768 |
| AUC | 0.801 | 0.802 | 0.798 | 0.8 | 0.805 |
| Model Performance on 24 Variables with SMOTE on Test Set | | | | | |
|  | **RF SMOTE** | **XGBOOST SMOTE** | **SVM SMOTE** | **Ensemble Model SMOTE** | **DNN SMOTE** |
| Sensitivity | 45.37% | 47.45% | 48.89% | 63.64% | 65.00% |
| Specificity | 91.30% | 89.49% | 89.15% | 82.37% | 77.66% |
| PPV | 70.23% | 67.13% | 67.07% | 62.01% | 56.82% |
| NPV | 78.70% | 79.02% | 79.41% | 83.36% | 83.07% |
| Accuracy | 77% | 76.40% | 76.61% | 76.54% | 73.72% |
| Kappa statistic | 0.4061 | 0.4017 | 0.4109 | 0.4568 | 0.4105 |
| AUC | 0.805 | 0.799 | 0.786 | 0.812 | 0.794 |
| Model Performance on 24 Variables with SMOTE on External Validation Set | | | | | |
|  | **RF SMOTE** | **XGBOOST SMOTE** | **SVM SMOTE** | **Ensemble Model SMOTE** | **DNN SMOTE** |
| Sensitivity | 47.75% | 47.01% | 43.35% | 63.47% | 61.69% |
| Specificity | 89.84% | 89.12% | 90.79% | 81.09% | 80.87% |
| PPV | 56.66% | 54.56% | 56.69% | 48.27% | 47.27% |
| NPV | 86.08% | 85.81% | 85.22% | 88.87% | 88.36% |
| Accuracy | 80.69% | 79.96% | 80.47% | 77.26% | 76.69% |
| Kappa statistic | 0.3986 | 0.3803 | 0.3731 | 0.4001 | 0.3834 |
| AUC | 0.795 | 0.791 | 0.784 | 0.8 | 0.791 |

**Table 6.** 14 Variable Logistic Regression Model Performance on Test and Validation Sets

| Model Performance on 14 Variables with Majority Class Undersampling on Test and External Validation Sets | | |
| --- | --- | --- |
|  | **Logistic Regression Under – Test Set** | **Logistic Regression Under – External Validation Set** |
| Sensitivity | 66.11% | 65.79% |
| Specificity | 74.71% | 76.30% |
| PPV | 54.17% | 43.55% |
| NPV | 82.98% | 88.92% |
| Accuracy | 72.03% | 74.01% |
| Kappa statistic | 0.3849 | 0.3554 |
| AUC | 0.78 | 0.78 |
| Model Performance on 14 Variables with SMOTE on Test and External Validation Sets | | |
|  | **Logistic Regression SMOTE – Test Set** | **Logistic Regression SMOTE – External Validation Set** |
| Sensitivity | 56.67% | 54.03% |
| Specificity | 81.61% | 82.37% |
| PPV | 58.22% | 46.00% |
| NPV | 80.64% | 86.57% |
| Accuracy | 73.84% | 76.2% |
| Kappa statistic | 0.3856 | 0.3424 |
| AUC | 0.78 | 0.78 |

**Table 7.** 14 Variable Deep Neural Network Performance on Test and Validation Sets Using MICE Imputation

| Model Performance on 14 Variables with Majority Class Undersampling on Test and External Validation Sets | | |
| --- | --- | --- |
|  | **DNN Under – Test Set** | **DNN Under – External Validation Set** |
| Sensitivity | 68.70% | 64.18% |
| Specificity | 72.01% | 74.42% |
| PPV | 52.62% | 41.10% |
| NPV | 83.58% | 88.20% |
| Accuracy | 70.98% | 72.19% |
| Kappa statistic | 0.3756 | 0.3209 |
| AUC | 0.774 | 0.76 |
| Model Performance on 14 Variables with SMOTE on Test and External Validation Sets | | |
|  | **DNN SMOTE – Test Set** | **DNN SMOTE – External Validation Set** |
| Sensitivity | 66.29% | 61.00% |
| Specificity | 73.58% | 76.11% |
| PPV | 53.15% | 41.52% |
| NPV | 82.84% | 87.53% |
| Accuracy | 71.31% | 72.82% |
| Kappa statistic | 0.3734 | 0.3174 |
| AUC | 0.768 | 0.752 |

**Figure 2.** Variable Correlations – Principal Components Analysis
